# Prospective observational cohort study of patients with weaning failure admitted to a specialist weaning, rehabilitation and home mechanical ventilation centre

**DOI:** 10.1136/bmjopen-2015-010025

**Published:** 2016-03-15

**Authors:** Denise Mifsud Bonnici, Thomas Sanctuary, Alex Warren, Patrick B Murphy, Joerg Steier, Philip Marino, Hina Pattani, Ben C Creagh-Brown, Nicholas Hart

**Affiliations:** 1 Lane Fox Respiratory Unit, Guy's and St Thomas’ NHS Foundation Trust, London, UK; 2 GKT School of Medical Education, King's College London, London, UK; 3 Faculty of Life Sciences and Medicine, King's College London, London, UK; 4 Intensive Care Unit, Royal Surrey County Hospital, Guildford, Surrey, UK; 5 Surrey Perioperative Anaesthesia and Critical care collaborative research group (SPACeR), Faculty of Health and Medical Sciences, University of Surrey, Guildford, Surrey, UK; 6 Lane Fox Clinical Respiratory Physiology Research Centre, Guy's and St Thomas’ NHS Foundation Trust, London, UK

**Keywords:** THORACIC MEDICINE

## Abstract

**Objectives:**

According to National Health Service England (NHSE) specialist respiratory commissioning specification for complex home ventilation, patients with weaning failure should be referred to a specialist centre. However, there are limited data reporting the clinical outcomes from such centres.

**Setting:**

Prospective observational cohort study of patients admitted to a UK specialist weaning, rehabilitation and home mechanical ventilation centre between February 2005 and July 2013.

**Participants:**

262 patients admitted with a median age of 64.2 years (IQR 52.6–73.2 years). 59.9% were male.

**Results:**

39.7% of patients had neuromuscular and/or chest wall disease, 21% were postsurgical, 19.5% had chronic obstructive pulmonary disease (COPD), 5.3% had obesity-related respiratory failure and 14.5% had other diagnoses. 64.1% of patients were successfully weaned, with 38.2% weaned fully from ventilation, 24% weaned to nocturnal non-invasive ventilation (NIV), 1.9% weaned to nocturnal NIV with intermittent NIV during the daytime. 21.4% of patients were discharged on long-term tracheostomy ventilation. The obesity-related respiratory failure group were most likely to wean (relative risk (RR) for weaning success=1.48, 95% CI 1.35 to 1.77; p<0.001), but otherwise weaning success rates did not significantly vary by diagnostic group. The median time-to-wean was 19 days (IQR 9–33) and the median duration of stay was 31 days (IQR 16–50), with no difference observed between the groups. Weaning centre mortality was 14.5%, highest in the COPD group (RR=2.15, 95% CI 1.19 to 3.91, p=0.012) and lowest in the neuromuscular and/or chest wall disease group (RR=0.34, 95% CI 0.16 to 0.75, p=0.007). Of all patients discharged alive, survival was 71.7% at 6 months and 61.8% at 12 months postdischarge.

**Conclusions:**

Following NHSE guidance, patients with weaning delay and failure should be considered for transfer to a specialist centre where available, which can demonstrate favourable short-term and long-term clinical outcomes.

**Strengths and limitations of this study**

- This study reports the clinical outcomes of a large cohort of patients with weaning failure admitted to a specialist weaning and rehabilitation centre.
- Details of clinical outcomes including time-to-wean, weaning success, weaning centre mortality and 1 year survival based on primary diagnostic case mix were included.
- The data describing the total duration of invasive ventilation are incomplete; however, all patients included met internationally accepted criteria for definition of weaning failure prior to admission.

## Introduction

The majority of patients admitted to the intensive care unit (ICU) requiring invasive mechanical ventilation can be weaned promptly on resolution and reversal of the primary insult. However, 31% of patients fail their initial spontaneous breathing trial (SBT) and require up to three SBTs, or up to 7 days from the first SBT, to achieve successful weaning with 7% of patients remaining ventilated longer than 1 week.^1^ Although there are emerging strategies to identify such patients at an early stage of critical illness,^2^ these patients with weaning failure who require prolonged mechanical ventilation have a greater ICU mortality, even after adjustment of confounding variables.^3^ ^4^ Increasingly, specialist weaning, rehabilitation and home ventilation centres are emerging to treat these patients, and the number of patients transferred to such units has been steadily growing in the USA^3^ and Europe,^4^ with variable outcomes reported.^5–16^

We report the outcome data of a national specialist weaning, rehabilitation and home mechanical ventilation unit in a London university teaching hospital, which since 1995 has taken referrals from ICUs across England and Wales. We have previously reported on costs and survival,^13^ but with National Health Service England (NHSE) specialist commissioning of complex home ventilation services,^17^ it is now essential for all such units to collect, analyse and publish their data, so that referral patterns, patient case mix and clinical outcome data can be benchmarked both nationally and internationally.

## Methods

### Defined clinical cohort

Data were prospectively collected from patients admitted to a UK specialist weaning, rehabilitation and home mechanical ventilation centre between February 2005 and July 2013. The study was conceived as a service evaluation and therefore assessment by the local research ethics committee was not required, but all data were anonymised. Patients were excluded if (1) they were receiving invasive tracheostomy ventilation, but were admitted for stabilisation and enhancement of the community care package to facilitate discharge to home or nursing home rather than admission for an attempt at weaning (eg, high spinal cord injury, catastrophic brain injury) or (2) they had been successfully weaned from invasive tracheostomy ventilation at the referring ICU and transferred for rehabilitation and establishment of home non-invasive ventilation (NIV). Weaning failure was defined as a patient transferred for weaning but deemed unsuitable following completion of a comprehensive multidisciplinary assessment in the weaning centre and all further weaning attempts discontinued. In essence, this was defined as patients requiring invasive ventilation at discharge or a time of death if they died in the centre. Weaning success was defined as a patient being liberated from invasive respiratory support at the time of discharge from the weaning centre.

### Data collection tool

Data were prospectively collected from a bespoke electronic medical records and chart system, including all clinical notes and medical discharge summaries (ICIP Carevue, Philips, Eindhoven, The Netherlands), from referral letters and the hospital electronic patient records system. Data were also collected from the referring hospital and the general practitioner by telephone and letter. The primary data record included age on admission, gender, primary diagnostic category, referring hospital, date of admission, date weaned from invasive mechanical ventilation, date of discharge, weaning outcome, discharge destination and date of death (if applicable). The secondary data record included time-to-wean (calculated as days between admission to the weaning and rehabilitation centre and removal of tracheostomy), length-of-stay (days between admission to and discharge from the weaning and rehabilitation centre) and postcentre discharge survival rate (proportion of patients alive from date of discharge from the weaning and rehabilitation centre up to 12 months postdischarge).

### Primary diagnostic groups

The primary diagnosis implicated in causing chronic respiratory failure and prolonged mechanical ventilation were classified into five diagnostic groups based on aetiology including (1) neuromuscular disorders and chest wall disease; (2) chronic obstructive pulmonary disease (COPD); (3) postsurgical; (4) obesity-related respiratory failure (ORRF), including obstructive sleep apnoea and/or obesity hypoventilation syndrome; and (5) other causes of chronic respiratory failure. The primary diagnosis was established at the weekly multidisciplinary meeting.

### Clinical outcome set

All patients received a bespoke weaning plan with twice daily medical reviews with the plan developed at the once weekly multidisciplinary weaning team meeting. We use a specific, non-protocolised approach to these complex weaning patients.^18^ The core clinical outcome set included rates of weaning success and weaning centre mortality, time-to-wean, and survival rate in the first 12 months after hospital discharge. Weaning outcomes were further classified into the following categories (1) self-ventilating day and night; (2) weaned onto nocturnal NIV; (3) weaned onto nocturnal NIV with intermittent daytime NIV use; (4) tracheostomy ventilation dependent (weaning failure); and (5) death within the weaning and rehabilitation centre which included patients weaned from invasive ventilation, but died prior to discharge from hospital.

#### Statistical analysis

Data were analysed using the Statistical Program for the Social Sciences (SPSS) V.22 (SPSS Inc, Chicago, Illinois, USA). Non-parametric data were expressed as median and IQR and compared using the Mann-Whitney U test or the Kruskal-Wallis test. Proportions were compared using the χ^2^ test for association or trend or Fisher's exact test as appropriate. Relative risk (RR) ratios were calculated using MedCalc (MedCalc.org, Ostend, Belgium) and we have reported the likelihood of successful weaning and death. Kaplan-Meier analysis was used to assess survival, with the log-rank test used to compare between groups. Statistical significance was accepted as a p value <0.05.

## Results

### Demographics

During the study period, 262 patients were admitted. The median age was 64.2 years (IQR=52.6–73.2). In total, 59.9% of patients were male (n=157), and 40.1% were female (n=105). In total, 72.5% of patients (n=190) were admitted as tertiary referrals from other hospitals, and 27.5% (n=72) were referred internally from the hospital ICU.

### Primary diagnostic groups

Neuromuscular and chest wall disease were the largest primary diagnostic category (n=105, 39.7%). The second largest diagnostic group was postsurgical (n=55, 21.0%). The third largest group was COPD (n=51, 19.5%) followed by the other group (n=38, 14.5%), and the ORFF group were the fewest in number (n=14, 5.3%). These data, detailing the primary diagnoses recorded at admission to our unit within patients from each primary diagnostic group, are shown in table 1.

**Table 1:** Admission demographic and diagnosis of 262 patients admitted to a specialist weaning and rehabilitation centre

Factor	Median (IQR) or n (%)
Age	64.2 (52.6–73.6)
Male gender	157 (59.9%)
Tertiary admission	190 (72.5%)
Neuromuscular/chest wall disease	105 (39.7%)
Motor neuron disease	17 (6.5%)
Structural chest wall disease	16 (6.1%)
Guillain-Barré syndrome	13 (5.0%)
Muscular dystrophy	13 (5.0%)
Myotonic dystrophy	11 (4.2%)
Spinal cord injury	6 (2.3%)
Isolated postcritical care neuromyopathy*	5 (1.9%)
Previous poliomyelitis	3 (1.1%)
Myasthenic syndrome	3 (1.1%)
Postsurgery	55 (21.0%)
Cardiothoracic	29 (11.1%)
Upper gastrointestinal	7 (2.7%)
Vascular surgery	6 (2.3%)
ENT/maxillofacial	5 (1.9%)
Lower gastrointestinal	4 (1.5%)
COPD	51 (19.5%)
Obesity-related respiratory failure	14 (5.3%)
Other diagnosis	38 (14.5%)
Interstitial lung disease	10 (3.8%)
Chronic respiratory infection	7 (2.7%)
Stroke	5 (1.9%)
Malignant disease	3 (1.1%)

Diagnoses with fewer than three patients admitted in this cohort are not given in this table.

*Five patients had no clear aetiology of weaning failure but had confirmed isolated postcritical care neuromyopathy without coexisting neurological disease.

COPD, chronic obstructive pulmonary disease; ENT, ear nose and throat.

### Weaning outcome by diagnostic group

**Figure 1:**
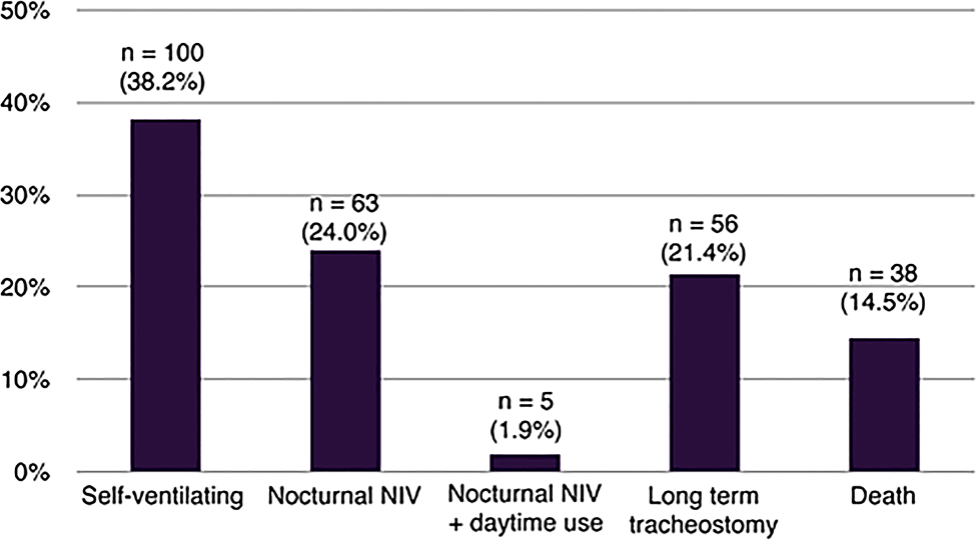
Clinical outcome of patients admitted to a specialist weaning and rehabilitation centre. NIV, non-invasive ventilation.

Weaning success was observed in 64.1% of patients (n=168). The most common outcome was successful weaning to self-ventilation (n=100, 38.2%), followed by weaning to nocturnal NIV only (n=63, 24.0%). A small number of patients (n=5, 1.9%) were discharged requiring NIV both nocturnally and intermittently during the day. In total, 21.4% of patients (n=56) were discharged on permanent tracheostomy ventilation. The weaning outcomes are shown in figure 1 and table 2.

**Table 2:** Weaning outcome by primary diagnostic group

n (%)	All patients n=262 (%)	NMD-CWD n=104 (%)	COPD n=51 (%)	Postsurgical n=55 (%)	ORRF n=14 (%)	Other n=38 (%)
Self-ventilating	100 (38.2)	29 (27.9)	24 (47.1)	27 (49.1)	6 (42.9)	14 (36.8)
Nocturnal NIV	63 (24.0)	35 (33.7)	9 (17.6)	8 (14.5)	6 (42.9)	5 (13.2)
Nocturnal NIV and daytime use	5 (1.9)	1 (1.0)	0 (0.0)	2 (3.6)	1 (7.1)	1 (2.6)
Long-term tracheostomy	56 (21.4)	32 (30.8)	5 (9.8)	8 (14.5)	0 (0.0)	11 (28.9)
Death	38 (14.5)	7 (6.7)	13 (25.5)	10 (18.2)	1 (7.1)	7 (18.4)
Total weaned	168 (64.1)	65 (62.5)	33 (64.7)	37 (67.3)	13 (92.9)	20 (52.6)

COPD, chronic obstructive pulmonary disease; CWD, chest wall disease; NIV, non-invasive ventilation; NMD, neuromuscular disease; ORRF, obesity-related respiratory failure.

There was no significant difference in weaning success rates observed between patients in the neuromuscular-chest wall disease (p=0.659), COPD (p=0.922) or postsurgical (p=0.571) diagnostic groups. The other diagnostic group demonstrated the lowest successful weaning rate at 52.6% (n=20), though this was also not significant. Conversely, 92.9% of patients with a primary diagnosis of ORRF were weaned successfully (RR for successful weaning=1.48, 95% CI 1.35 to 1.77, p<0.001; table 3).

**Table 3:** Weaning success

Factor	RR for weaning success	95% CI	p Value
Male gender	0.89	0.75 to 1.07	0.212
Outside hospital referral	0.97	0.80 to 1.19	0.808
NMD/CWD	0.96	0.79 to 1.16	0.659
COPD	1.01	0.81 to 1.27	0.922
Postsurgical	1.06	0.86 to 1.31	0.571
ORRF	1.48	1.25 to 1.77	<0.001

COPD, chronic obstructive pulmonary disease; CWD, chest wall disease; NMD, neuromuscular disease; ORRF, obesity-related respiratory failure; RR, relative risk.

The likelihood of weaning success, by primary diagnostic group, is shown in table 3.

### Mortality by diagnostic group

Overall, weaning centre mortality was 14.5% (n=38). There was a difference in mortality between the diagnostic categories with patients with COPD demonstrating an increased weaning centre mortality (RR for death=2.15, 95% CI 1.19 to 3.91, p=0.012). Conversely, the neuromuscular-chest wall disease group had a weaning centre mortality of only 6.7% (RR for death=0.34, 95% CI 0.16 to 075, p=0.007), despite having a comparable rate of weaning success. This was reflected in the fact that the patients with neuromuscular/chest wall disease were more frequently discharged on long-term tracheostomy ventilation (n=32, 30.8%; p=0.004). The obese patients with ORRF had a comparably low mortality with the neuromuscular and chest wall disease group (table 4).

**Table 4:** Mortality within the weaning and rehabilitation centre

Factor	RR for death	95% CI	p Value
Male gender	1.14	0.62 to 2.11	0.661
Outside hospital referral	0.73	0.39 to 1.35	0.311
NMD-CWD	0.34	0.16 to 0.75	0.007
COPD	2.15	1.19 to 3.91	0.012
Postsurgical	1.34	0.70 to 2.60	0.378
OSA-OHS	0.48	0.07 to 3.24	0.450

COPD, chronic obstructive pulmonary disease; CWD, chest wall disease; NMD, neuromuscular disease; OHS, obesity hypoventilation syndrome; OSA, obstructive sleep apnoea; RR, relative risk.

### Time-to-wean

The median time-to-wean was 19 days (IQR=9–33 days; range 0–121 days). There was no difference observed between the diagnostic groups (p=0.478). As expected, there was substantial variation between patients. Of the 168 patients who were weaned from invasive ventilation, 51 patients (29.6%) had a time-to-wean greater than 30 days.

### Length-of-stay

The median length-of-stay was 31 days (IQR 16–50 days; range 1–364 days) with no difference observed between the diagnostic groups (p=0.242). Of the patients who were weaned from invasive mechanical ventilation, 51 patients (29.6%) were an inpatient in the weaning and rehabilitation centre for more than 60 days and 24 patients (14.0%) were an inpatient more than 100 days. The time-to-wean and length-of-stay by primary diagnostic group are shown in table 5.

**Table 5:** Time-to-wean and length-of-stay in the weaning and rehabilitation centre of patients weaned from invasive mechanical ventilation

(n)	Time-to-wean (days)		Length-of-stay (days)	
	Median (IQR)	Range	Median (IQR)	Range
All patients (n=168)	19 (9–33)	0–121	8 (5–13)	1–364
NMD-CWD (n=65)	20.5 (10–36)	1–112	9 (5–17)	1–57
COPD (n=33)	16 (10–28)	1–79	5 (4–10)	1–22
Postsurgical (n=37)	24.5 (7.75–37.25)	0–51	13 (4–16.75)	2–89
ORRF (n=13)	14 (9.5–34–5)	1–108	8 (5.5–13.5)	1–364
Other (n=20)	19.5 (9–31.5)	1–121	7 (4.25–13.75)	1–145

COPD, chronic obstructive pulmonary disease; CWD, chest wall disease; NMD, neuromuscular disease; ORRF, obesity-related respiratory failure.

### Discharge from the weaning, rehabilitation and home mechanical ventilation centre

**Figure 2:**
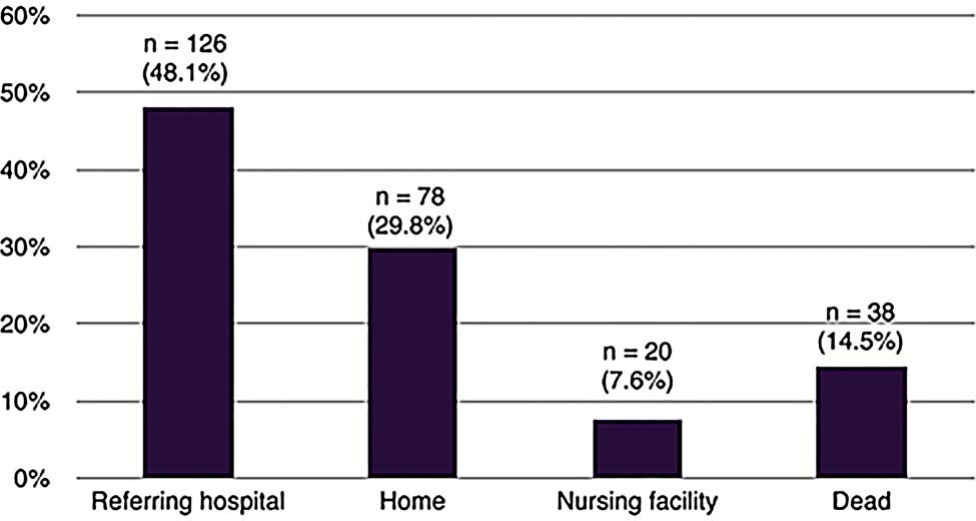
Discharge destination of patients admitted to a specialist weaning and rehabilitation centre.

The major proportion of patients discharged alive from the specialist centre returned to the referring hospital for further rehabilitation (n=126, 48.1%) with 78 patients (30.0%) discharged directly to home and 20 (7.7%) discharged directly to a nursing home or long-term residential care facility. Discharge outcome data for the cohort are shown in figure 2.

### Postdischarge mortality

**Figure 3:**
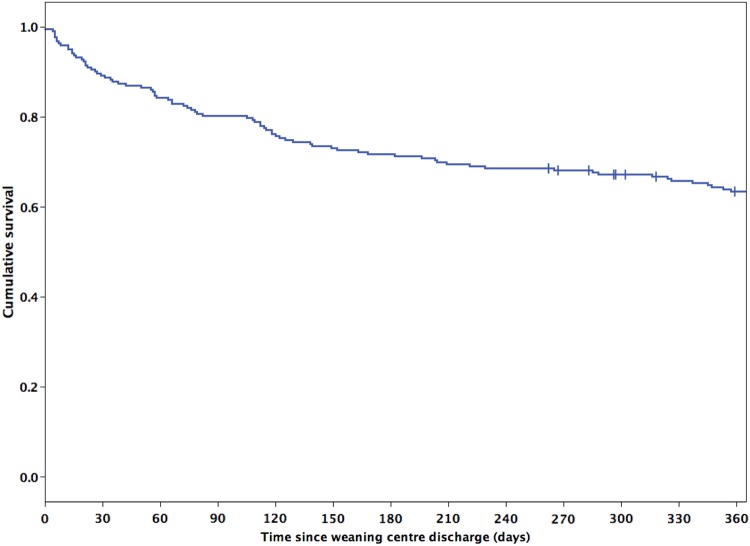
Twelve-month Kaplan-Meier survival curve for all patients discharged alive; n=223. | = censored observation (n=9).

**Figure 4:**
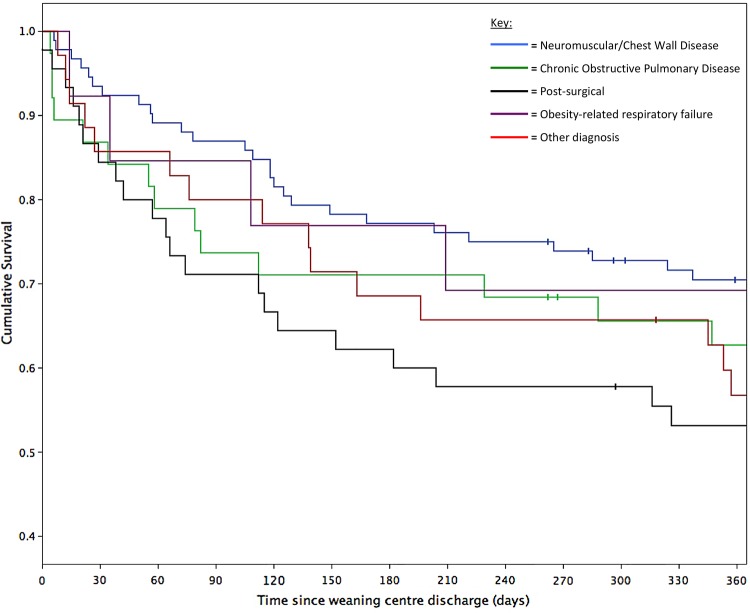
Twelve-month Kaplan-Meier survival curve separated by diagnostic group, n=223, | = censored observation (n=9).

Of the 224 patients discharged alive, 6-month survival status was available for 223 (99.6%) and 12-month survival status for 215 (96.0%). Cumulative survival at 6 months was 71.7% (n=160) and at 12 months was 61.8% (n=133; figure 3). There was no difference in mortality between the primary diagnostic groups during the 12 months postdischarge (log rank p=0.202; figure 4). Survival at 12 months was 70.5% (n=61) in the neuromuscular-chest wall disease group, 69.2% (n=9) in the ORRF group, 62.7% (n=22) in the COPD group, 56.8% (n=19) in the other diagnostic group and 53.2% (n=23) in the postsurgical group.

## Discussion

In the present study, the majority of patients with weaning failure admitted had neuromuscular and chest wall disease as the primary diagnostic group. Weaning success was observed in 65% of the cohort with weaning failure and death observed in 21% and 15%, respectively. There was no difference in weaning success across the different diagnostic groups with a median time-to-wean following admission to the specialist centre of 19 days. Eighty-five per cent of patients survived to hospital discharge with 62% of those discharged alive surviving for 1 year following discharge from the weaning and rehabilitation centre.

This is the largest observational cohort study reporting the clinical outcome of complex patients transferred to a specialist weaning, rehabilitation and home mechanical ventilation centre. These data are timely, and indeed important, with the imminent implementation of the NHSE specialist respiratory commissioning specification detailing complex home ventilation.^17^ A requirement of this specification will be for specialist centres to report a clinical outcome set, including referral pattern, weaning success, time-to-wean, weaning failure and weaning centre mortality, which can then be benchmarked both nationally and internationally.

### Limitations of the study

Although the 1 year follow-up data were wholly acceptable with only nine patients lost to follow-up at 12 months, the data from the referring hospitals were incomplete, and therefore the study was unable to comment on the total time of invasive ventilation as the time of receiving invasive ventilation in the ICU prior to arriving at the weaning and rehabilitation centre was not complete and reliable. However, this can be balanced by the admission criteria to the centre which includes invasive ventilation for greater than 28 days, and so we are confident that the patients all had weaning failure, by definition,^1^ ^17^ prior to admission. In addition, the present study was unable to comment on the number, and proportion, of patients referred to the centre but who were not admitted. As expected, patients were not admitted if they were deemed unweanable, died awaiting transfer or were weaned locally in the referring ICU. This may have resulted in a selection bias which would be expected to enhance weaning outcome. These important clinical data will need to be included in the NHSE core outcome set for patients with weaning failure.

### Clinical outcome set for weaning

The clinical outcomes of weaning success, time-to-wean and weaning centre mortality have improved compared with the data we published in our smaller 3-year cohort study.^13^ Interestingly, the proportion of the primary diagnostic groups have not changed since the previous report, with neuromuscular and chest wall disease still predominating. This reflects the historical background of the centre for the management of patients with previous poliomyelitis syndrome and postpolio syndrome^19^ ^20^ and, recently, for patients with inherited muscle disorders, such as Duchenne muscular dystrophy.^21^ ^22^ In the current study, patients with neuromuscular and chest wall disease had the lowest weaning centre mortality, despite a comparable rate of successful weaning with the other groups, and thus had the highest incidence of discharge requiring long-term tracheostomy ventilation. A patient with COPD had the highest weaning centre mortality rate. This represents a change from the earlier reported cohort, which found that postsurgical patients were more likely to die during the weaning process.^13^ While patients with ORRF formed a small part of this cohort, this study has confirmed previous data^23^ ^24^ that the obese group are likely to successfully wean from invasive ventilation and indeed have a 12-month survival similar to the other groups. Notably, the time-to-wean and length-of-stay data demonstrated large variation, which reflects the challenges of managing patients with weaning failure.

### Discharge destination

The current study observed that a smaller proportion of patients were discharged to home following successful weaning, compared with the previous data,^13^ with more patients discharged back to the referring hospital. This is an interesting observation and reflects the increasing challenges of the multidisciplinary team at specialist tertiary centres liaising with multiple clinical commissioning group continuing healthcare teams and social services teams when the patient is from a distant geographical location. The effect of this is that patients remain in the specialist centre for an extended length-of-stay, a median of 31 days in the current study when the time-to-wean was 19 days, which limits the admission of further patients for assessment and review. Indeed, the adverse effect of delayed discharge from the weaning and rehabilitation centre has resulted in a reduced capacity with repatriation as an expeditious alternative to complex community discharge planning.

### Survival and follow-up postdischarge from the weaning and rehabilitation centre

Although discharge mortality was low at 15%, mortality during the first 6 months postdischarge was twice that of the second 6-month period postdischarge, albeit there was no difference between the primary diagnostic groups. This is important to acknowledge and highlight that there are specific patients that are at a high risk of death postdischarge. However, even in post hoc analysis when we considered the patients in expected clinical prognostic groups, rather than the diagnostic groups, we could not identify a subgroup of patients at highest risk. Indeed, in subgroup analysis, we separated the patients as slowly progressive disease (eg, Duchenne muscular dystrophy, postpolio), patients with conditions in recovery (eg, Guillain-Barré syndrome, ICU-acquired weakness^25^) and frail patients with rapidly progressive disease at high risk of subsequent decline (eg, motor neuron disease, COPD), but there was no difference in cumulative survival by disease prognosis (log rank p=0.057). Clinicians will need to follow all patients postdischarge and determine the individual trajectory of recovery or decline, with a focus on the first 6 months following discharge. More observational data detailing the characteristics of the patients who recover and those who decline are required to stratify the patients postdischarge and to develop the optimum clinical management strategy. This lack of stratification may have influenced, in part, the lack of evidence currently to support the development of post-ICU clinics.^26^ ^27^

### Benchmarking

Developing a clinical outcome set for weaning and rehabilitation centres is essential. However, just as comparison of ICU mortality outcomes requires defining the standardised mortality ratio to ensure the comparative analysis is worthwhile, we must develop such an approach for the weaning and rehabilitation centres to accommodate the patient case mix. Although weaning centre survival and weaning success and 12-month postdischarge survival will be key outcome metrics, we need to consider these in the context of the primary diagnostic groups, subgroups of primary diagnostic groups and other factors, such as age and frailty, to identify those patients which are at high risk of clinical deterioration within 6 months of discharge. This will allow national and international benchmarking of such centres and explain, in part, the wide differences reported of both weaning success and 1-year mortality, which currently range from 56% to 95% and 8% to 78%, respectively, from both specialist and non-specialist centres.^4–6^ ^8^ ^13^ ^15^ ^16^

### Specialist weaning, rehabilitation and home ventilation centres in the UK

There are currently few weaning, rehabilitation and home ventilation centres in the UK, despite a report from the UK Department of Health recommending an expansion of such specialist services with estimated cost savings in the region of 50% per patient per day compared with managing a patient in an ICU.^28^ The North of England study determined that patients with weaning failure occupied 1000 ICU bed days per year, which can be extrapolated to 12 500 ICU bed days annually across the UK.^29^ These data have been supported by recent UK data suggesting a reduction in ICU bed occupancy by up to 10% with the establishment of specialist weaning and rehabilitation centres.^30^ In addition, acute hospital trusts wholly benefit by the transfer of patients with weaning failure to specialist centres as the increased critical care bed capacity can be used to maintain the high-risk elective surgical activity for the local patients, which will generate income for the hospital.^31^ Of the 3991 adult critical care beds across England,^32^ only a small proportion of patients will experience weaning failure, and indeed these patients are spread across the 240 ICUs across England. It is highly unlikely that there will be a randomised controlled trial comparing the outcome of specialist weaning and rehabilitation centres, long-term acute care facilities and ICUs, and therefore strategies to accommodate bed capacity, case-mix and relevant clinical outcomes must be developed as part of the specification of the NHSE complex home ventilation programme. Indeed, these specialist units offer bespoke weaning and rehabilitation for a small number of patients where protocolisation, a common feature of ICU management, is disregarded in favour of a personalised care plan for these patients with complex weaning failure.^18^

## Conclusion

The current data demonstrate that, in our centre, two-thirds of patients with weaning failure can be successfully weaned with over three-quarters of patients surviving to hospital discharge. In accordance with NHSE specialist respiratory commissioning specification for complex home ventilation, weaning, rehabilitation and home mechanical ventilation centres must collect a minimum data set that represents a core clinical outcome set, including weaning success, time-to-wean, weaning centre mortality and 12-month mortality. Twelve-month survival did not vary across the primary diagnosis but further characterisation of these patients postdischarge is required. In future, specialist centres will need to report these data as part of both national and international benchmarking exercises.

## References

[1] Boles JM, Bion J, Connors A Weaning from mechanical ventilation. Eur Respir J 2007;29:1033–56. 10.1183/09031936.00010206 17470624

[2] Glover G, Connolly B, Di Gangi S An observational cohort study to determine efficacy, adherence and outcome of the early initiation of pressure support ventilation during mechanical ventilation. BMJ Open Respir Res 2014;1:e000028 10.1136/bmjresp-2014-000028 PMC4212705 25478179

[3] Kahn JM, Benson NM, Appleby D Long-term acute care hospital utilization after critical illness. JAMA 2010;303:2253–9. 10.1001/jama.2010.761 20530778 PMC3094575

[4] Confalonieri M, Gorini M, Ambrosino N Respiratory intensive care units in Italy: a national census and prospective cohort study. Thorax 2001;56:373–8. 10.1136/thorax.56.5.373 11312406 PMC1746048

[5] Hannan LM, Tan S, Hopkinson K Inpatient and long-term outcomes of individuals admitted for weaning from mechanical ventilation at a specialised ventilation weaning unit. Respirology 2013;18:154–60. 10.1111/j.1440-1843.2012.02266.x 22985330

[6] Schonhofer B, Euteneuer S, Nava S Survival of mechanically ventilated patients admitted to a specialised weaning centre. Intensive Care Med 2002;28:908–16. 10.1007/s00134-002-1287-5 12122529

[7] Dasgupta A, Rice R, Mascha E Four-year experience with a unit for long-term ventilation (respiratory special care unit) at the Cleveland Clinic Foundation. Chest 1999;116:447–55. 10.1378/chest.116.2.447 10453875

[8] Quinnell TG, Pilsworth S, Shneerson JM Prolonged invasive ventilation following acute ventilatory failure in COPD: weaning results, survival, and the role of noninvasive ventilation. Chest 2006;129:133–9. 10.1378/chest.129.1.133 16424423

[9] Heinemann F, Budweiser S, Jorres RA The role of non-invasive home mechanical ventilation in patients with chronic obstructive pulmonary disease requiring prolonged weaning. Respirology 2011;16:1273–80. 10.1111/j.1440-1843.2011.02054.x 21883681

[10] Rose L, Fraser IM Patient characteristics and outcomes of a provincial prolonged-ventilation weaning centre: a retrospective cohort study. Can Respir J 2012;19:216–20. 22679615 10.1155/2012/358265 PMC3418097

[11] Scheinhorn DJ, Hassenpflug MS, Votto JJ Post-ICU mechanical ventilation at 23 long-term care hospitals: a multicenter outcomes study. Chest 2007;131:85–93. 10.1378/chest.06-1081 17218560

[12] Stoller JK, Xu M, Mascha E Long-term outcomes for patients discharged from a long-term hospital-based weaning unit. Chest 2003;124:1892–9. 10.1378/chest.124.5.1892 14605065

[13] Pilcher DV, Bailey MJ, Treacher DF Outcomes, cost and long term survival of patients referred to a regional weaning centre. Thorax 2005;60:187–92. 10.1136/thx.2004.026500 15741433 PMC1747325

[14] Aboussouan LS, Lattin CD, Anne VV Determinants of time-to-weaning in a specialized respiratory care unit. Chest 2005;128:3117–26. 10.1378/chest.128.5.3117 16304251

[15] Latriano B, McCauley P, Astiz ME Non-ICU care of hemodynamically stable mechanically ventilated patients. Chest 1996;109:1591–6. 10.1378/chest.109.6.1591 8769516

[16] Gracey DR, Hardy DC, Naessens JM The Mayo Ventilator-Dependent Rehabilitation Unit: a 5-year experience. Mayo Clinic proceedings. Mayo Clinic 1997;72:13–19. 10.4065/72.1.13 9005279

[17] NHS England Specialised Respiratory Commissioning Group. 2013/14 NHS CONTRACT FOR RESPIRATORY: COMPLEX HOME VENTILATION (ADULT). http://www.england.nhs.uk/wp-content/uploads/2013/06/a14-respiratory-comp-home-vent.pdf (accessed Sep 2015).

[18] Creagh-Brown B, Steier J, Hart N Prolonged weaning. In: Stevens R, Hart N, Herridge M, eds. The legacy of critical care: a textbook of post ICU medicine. Oxford: Oxford University Press, 2014;Chap 49:559–71. doi:10.1093/med/9780199653461.001.0001 10.1093/med/9780199653461.001.0001

[19] Howard RS Poliomyelitis and the postpolio syndrome. BMJ 2005;330:1314–18. 10.1136/bmj.330.7503.1314 15933355 PMC558211

[20] Davidson AC, Auyeung V, Luff R Prolonged benefit in post-polio syndrome from comprehensive rehabilitation: a pilot study. Disabil Rehabil 2009;31:309–17. 10.1080/09638280801973206 18608421

[21] Parker AE, Robb SA, Chambers J Analysis of an adult Duchenne muscular dystrophy population. QJM 2005;98:729–36. 10.1093/qjmed/hci113 16135534

[22] Nicot F, Hart N, Forin V Respiratory muscle testing: a valuable tool for children with neuromuscular disorders. Am J Respir Crit Care Med 2006;174:67–74. 10.1164/rccm.200512-1841OC 16574932

[23] Duarte AG, Justino EBS, Bigler TMS Outcomes of morbidly obese patients requiring mechanical ventilation for acute respiratory failure. Crit Care Med 1997;35:732–7. 10.1097/01.CCM.0000256842.39767.41 17255878

[24] Akinnusi ME, Pineda LA, El Solh AA Effect of obesity on intensive care unit morbidity and mortality: a meta-analysis. Crit Care Med 2008;36:151–8. 10.1097/01.CCM.0000297885.60037.6E 18007266

[25] Connolly B, Thompson A, Douri A Exercise-based rehabilitation after hospital discharge for survivors of critical illness with intensive care unit-acquired weakness: a pilot feasibility trial. J Crit Care 2015;30:589–98. 25703957 10.1016/j.jcrc.2015.02.002 PMC4416081

[26] Connolly B, Douri A, Steier J A UK survey of rehabilitation following critical illness: implementation of NICE Clinical Guidance 83 (CG83) following hospital discharge. BMJ Open 2014;4:e004963 10.1136/bmjopen-2014-004963 PMC4025447 24833691

[27] Cuthbertson BH, Rattray J, Campbell MK The PRaCTICal study of nurse led, intensive care follow-up programmes for improving long term outcomes from critical illness: a pragmatic randomised controlled trial. BMJ 2009;339:b3723 10.1136/bmj.b3723 19837741 PMC2763078

[28] NHS Modernisation Agency Report. Critical care programme. Weaning and long term ventilation. London: NHS Modernisation Agency, 2002.

[29] Robson V, Poynter J, Lawler PG The need for a regional weaning centre, a one-year survey of intensive care weaning delay in the Northern Region of England. Anaesthesia 2003;58:161–5. 10.1046/j.1365-2044.2003.02964_1.x 12622105

[30] Lone NI, Walsh TS Prolonged mechanical ventilation in critically ill patients: epidemiology, outcomes and modelling the potential cost consequences of establishing a regional weaning unit. Crit Care 2011;15:R102 10.1186/cc10117 21439086 PMC3219374

[31] Seneff MG, Wagner D, Thompson D The impact of long-term acute-care facilities on the outcome and cost of care for patients undergoing prolonged mechanical ventilation. Crit Care Med 2000;28:342–50. 10708164 10.1097/00003246-200002000-00009

[32] Government Statistical Service. Monthly Critical Care Beds and Cancelled Urgent Operations. http://www.england.nhs.uk/statistics/wp-content/uploads/sites/2/2015/05/July-15-Monthly-SitRep-SPN.pdf (accessed Sep 2015).

